# Thermal Conductance of Copper–Graphene Interface: A Molecular Simulation

**DOI:** 10.3390/ma15217588

**Published:** 2022-10-28

**Authors:** Jiarui Zhu, Shuhui Huang, Zhongnan Xie, Hong Guo, Hui Yang

**Affiliations:** 1 State Key Laboratory of Nonferrous Metals and Processes, GRINM Group Co., Ltd., Beijing 100088, China; 2 GRIMAT Engineering Institute Co., Ltd., Beijing 101407, China; 3 General Research Institute for Nonferrous Metals, Beijing 100088, China

**Keywords:** thermal boundary conductance, Cu/graphene composites, molecular dynamics, composites interface

## Abstract

Copper is often used as a heat-dissipating material due to its high thermal conductivity. In order to improve its heat dissipation performance, one of the feasible methods is to compound copper with appropriate reinforcing phases. With excellent thermal properties, graphene has become an ideal reinforcing phase and displays great application prospects in metal matrix composites. However, systematic theoretical research is lacking on the thermal conductivity of the copper–graphene interface and associated affecting factors. Molecular dynamics simulation was used to simulate the interfacial thermal conductivity of copper/graphene composites, and the effects of graphene layer number, atomic structure, matrix length, and graphene vacancy rate on thermal boundary conductance (TBC) were investigated. The results show that TBC decreases with an increase in graphene layers and converges when the number of graphene layers is above five. The atomic structure of the copper matrix affects the TBC, which achieves the highest value with the (011) plane at the interface. The length of the copper matrix has little effect on the TBC. As the vacancy rate is between 0 and 4%, TBC increases with the vacancy rate. Our results present insights for future thermal management optimization based on copper matrix composites.

## 1. Introduction

Electronic devices are increasingly becoming more integrated, multi-functional, and smaller as a result of the rapid development of the electronics industry. The increment in power density per unit volume produces a great deal of heat, which needs to be quickly dissipated to avoid negative impacts on the efficiency and longevity of equipment [1,2,3]. By incorporating a reinforcement phase with a low density and low expansion coefficient into the conventional metallic heat dissipation material, a metal matrix composite possesses high thermal conductivity and a low expansion coefficient as a typical thermal management composite material [4,5]. Metal matrix composites (MMCs) have been used for a variety of industrial applications, especially thermal management devices and aerospace materials [6,7,8,9]. Copper is a good composite matrix due to its excellent thermal conductivity, high melting temperature (1083 °C), good chemical stability, and corrosion resistance [10]. By incorporating a suitable reinforcing phase, Cu-based MMCs provide the opportunity to customize copper’s metallic characteristics to satisfy the demands of thermal management. Some carbon materials, such as diamond, graphite, and graphene, are good candidates for the reinforcing phase [5,11,12,13].

Graphene has attracted the attention of many researchers due to its excellent thermal properties [14,15]. In experiments, many studies have been conducted on composite materials prepared with copper as a matrix and graphene as a reinforcing phase. Chu et al. enabled the in-plane thermal conductivity of graphene/copper composites to reach 525 W/mK by adding 35 vol% graphene nanoplatelet [16]. Gao et al. reported that the thermal conductivity of the composite reached a peak of 397 W/mK when the graphene oxide content reached 0.3 wt.% [17]. In another work, Omayma et al. added graphene nanosheets to Cu/WC-TiC-Co [18]. When the graphene nanosheet content increases from 0 to 1wt.%, the thermal conductivity of the composite increases from 190 W/mK to 351 W/mK. Enhancing the heat transfer between adjacent materials is the key to improving the thermal conductivity of composite materials, so the thermal boundary conductance (TBC) between materials cannot be ignored in the study of material thermal properties. Although previous experiments have demonstrated that graphene as a reinforcing phase can effectively improve the thermal conductivity of copper matrix composites, data on the copper/graphene interface are relatively scarce. Measuring TBC is an important method to characterize the interfacial thermal properties of different materials, and it is impacted by defects, atomic structure, and interfacial bonding strength [19,20,21]. Therefore, it is necessary to systematically study the TBC of Cu/graphene. Molecular dynamics have been widely applied to investigate the heat transport process across solid–solid interfaces. It has been shown that interfacial phonon scattering plays an important role in heat transport at the graphene/metal interface at the temperature range from 50 to 500 K [22]. Problems regarding phonon scattering can be well explored utilizing molecular dynamics.

In this work, we have studied the heat transfer characteristics and mechanism of the Cu/Gr interface using the nonequilibrium molecular dynamics (NEMD) method. Table 1 lists the studies of different metal matrix/graphene composites using the NEMD method. The effects of graphene layer number, Cu matrix atomic structure, and graphene vacancy rate on the TBC have been systematically studied. Meanwhile, the phonon coupling factor is used to explain the underlying mechanism.

## 2. Model and Methods

The large-scale atomic/molecular massively parallel simulator (LAMMPS) program is applied to carry out the molecular dynamics (MD) simulations [26,27,28]. Reliable forcefields are required to achieve accurate MD simulations. However, there is not yet a single forcefield that can be used to describe a system made of Cu and C. In this work, we adopted a hybrid method including the well-known exact forcefields, which describe the Cu–Cu and C–C interactions. To explain forcefields of copper atoms and carbon atoms, respectively, the embedded atom method (EAM) [29,30] and optimized AIREBO potentials [31,32] are used in this work. Meanwhile, the Lennard-Jones (LJ) function V(r_ij_) = 4ε[(σ/r_ij_)^12^ − (σ/r_ij_)^6^] with the values σ_Cu−Gr_ = 0.30825 nm and ε_Cu−Gr_ = 0.02578 eV describes the non-bonded van der Waals interactions between Cu and C atoms [33,34]. Based on these forcefield descriptions, the constructed models are first minimized to achieve equivalent states and then undergo the NEMD methods to calculate the TBC. Below are the details about model construction, NEMD methods, and underlying phonon spectra.

### 2.1. Model

**Figure 1:**
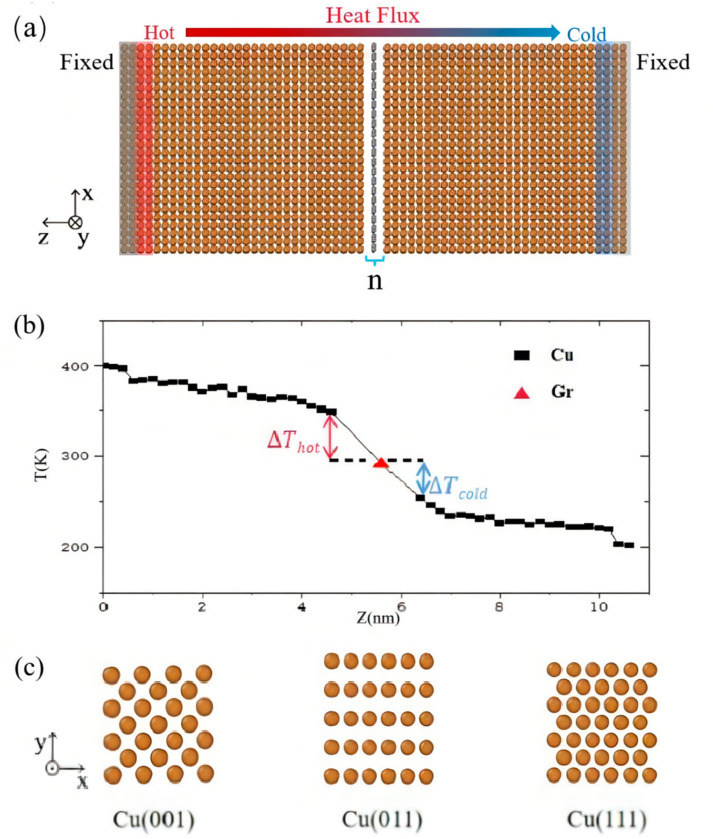
(**a**) Schematic of the NEMD method. (**b**) Temperature distribution of the copper/graphene model. The temperature differences near the hot and cold zones are denoted by ΔT_hot_ and ΔT_cold_, respectively. (**c**) Atomic structures of copper (100), (110), and (111) surfaces.

Figure 1a depicts the Cu/Gr composite model, which consists of two blocks of face-centered cubic (FCC) Cu crystals sandwiching one n-layer graphene block (*n* = 1–7). The value *n* here refers to the number of layers of graphene. L_x_, L_y_, and L_t_ stand for the Cu block’s length, breadth, and thickness, respectively. To guarantee tiny lattice mismatch strain imposed in the Cu/Gr interface structure, we chose different periods while similar model lengths for the copper and graphene models (Figure 1c). Graphene is subjected to tensile strains of 0.407% and 0.085% at two corresponding directions, respectively, in the simulation box, which is set as 4.69 nm in both the x- and y-directions. Table 2 lists the model parameters of different Cu crystal planes when the number of graphene layers *n* = 1. The interlayer gap between graphene layers is 0.34 nm, while the lamellar distance at the neighboring Cu/Gr layers is relaxed to 0.30 nm [35]. Two layers of Cu atoms on each side in the z direction must be fixed. Meanwhile, two layers of atoms adjacent to the fixed layers serve as the heat source (hot) and heat sink (cold) thermostats, respectively.

### 2.2. NEMD Method

The NEMD method establishes a non-equilibrium thermal conductivity process by exerting the disturbance of the system and then calculates the thermal conductivity through Fourier’s law of thermal conductivity [36,37]. As shown in Figure 1b, the NEMD method involves placing a cold source and a heat source at both the material’s ends, adding heat to the heat source, subtracting equal amount of heat from the cold source, and, finally, calculating the heat transfer and temperature gradient as a result. Simulations were run with periodic boundary conditions carried out along the x and y (in-plane) directions and free boundary conditions in the z (out-of-plane) direction in order to obtain the TBC based on the NEMD method. Using the Langevin thermostat, we adjusted the heat and cold resources to temperatures of 400 K and 200 K, respectively [38]. The imposed temperature difference activates the process of directional heat transfer of the system. A temperature gradient was produced after 0.5 ns, with each component time-independent. Each block of the Cu model was partitioned into 26 equivalent slabs along z direction (the heat flux) to evaluate the temperature distribution (Figure 1b), and the velocity data during the last 1 ns were collected for vibrational analysis. The energy change over time (heat flux) can be calculated as follows: J = dΔE(t)/dt; here, ΔE means the energy change. The TBC can be obtained from G = J/AΔT; here, A indicates the cross-section area of heat transfer. Here, we calculate T by averaging the two temperature differences that occurred adjacent to the Cu/Gr interfaces’ heat source and heat sink.

### 2.3. Phonon Spectra

We studied the vibrational density of states (VDOS) across the interfaces to clarify the process of interfacial heat transfer. First, the velocity autocorrelation function (Vacf) [39] can be obtained from Lammps by analyzing the velocities of each atom in different directions:

(1) $$\mathrm{Vacf}\left(t\right)=\frac{1}{N}\overset{N}{\underset{i=1}{\sum }}\langle v_{i}\left(0\right)v_{i}\left(t\right)\rangle ,$$

Then, the VDOS is calculated through the Fourier transform of the Vacf:

(2) $$\mathrm{VDOS}\left(\omega \right)=\int_{-\infty }^{\infty }e^{i\omega t}\mathrm{VACF}\left(t\right)\mathrm{dt},$$

The out-of-plane phonon dominates the thermal transfer process between the interfacial graphene and copper layers, and the low-frequency zone is primarily responsible for phonon coupling. S, the phonon spectrum parameter, defines the overlap as:

(3) $$S=\frac{\int_{0}^{\infty }P_{\mathrm{Gr}}\left(\omega \right)P_{\mathrm{Cu}}\left(\omega \right)d\omega }{\int_{0}^{\infty }P_{\mathrm{Gr}}\left(\omega \right)d\omega \times \int_{0}^{\infty }P_{\mathrm{Cu}}\left(\omega \right)d\omega },$$

where P_Gr_(ω) and P_Cu_(ω) denote the phonon spectra at frequency ω of graphene and Cu atoms at the interface, respectively.

## 3. Results and Discussion

### 3.1. Effect of Graphene Layer Number

**Figure 2:**
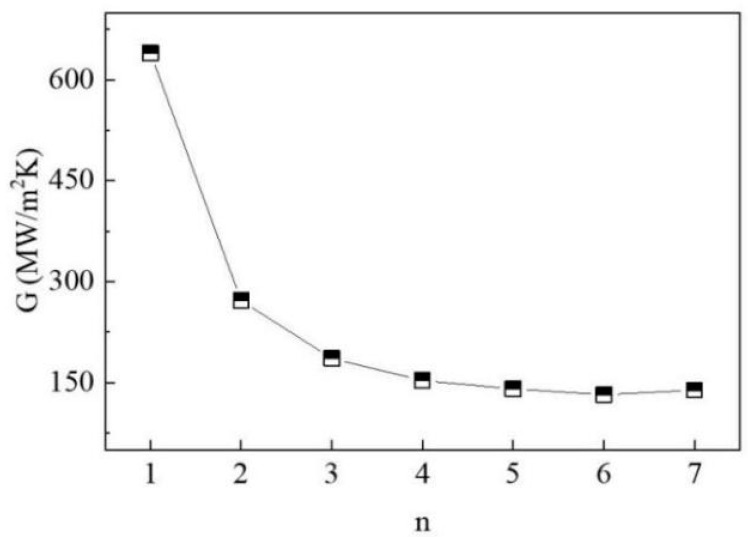
Effect of graphene layer number on TBC.

We used the Cu(001)/Gr interface model to study the effect of graphene layer number on TBC. The simulations were performed at 300 K through the NEMD approach, and Figure 1a depicts the associated schematic model. The two adjacent atom layers were set as cold (heat sink) and hot (heat source) thermostats, respectively, and both ends were fixed. As Figure 2 displays, the TBC reaches a maximum value of 640.19 MW/m^2^K when *n* = 1. With the increase in n, the TBC gradually decreases until it converges at *n* = 5. There is a reduction of 78% from 640.19 MW/m^2^K (*n* = 1) to 140.44 MW/m^2^K (*n* = 5).

**Figure 3:**
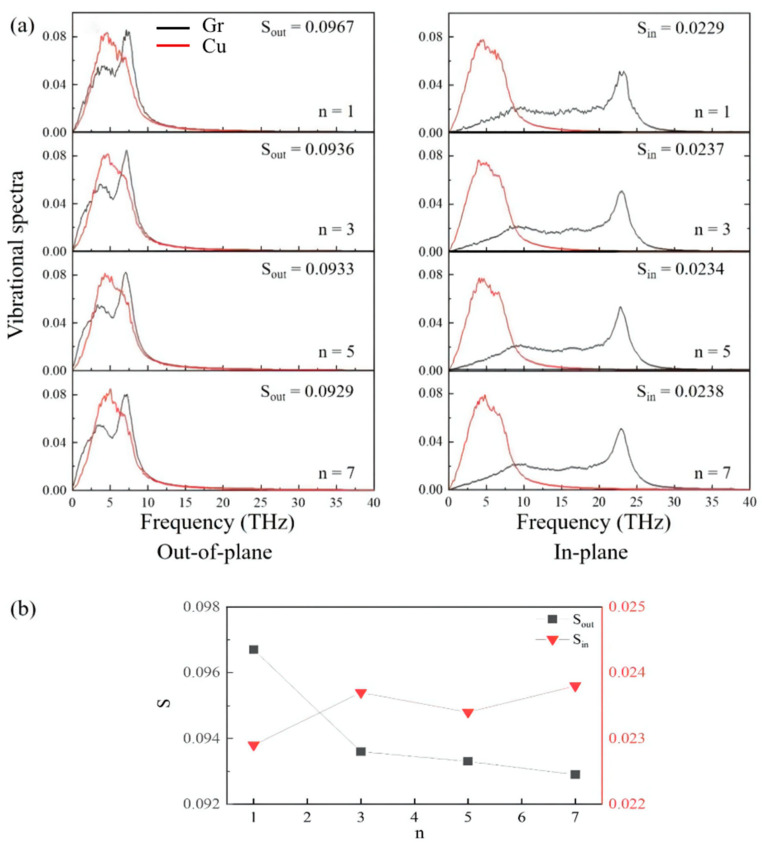
(**a**) Vibrational spectra of different modes of Cu and graphene atoms at the interface; n depicts the number of graphene layers. (**b**) The overlap concentration of phonon spectrum S with different layers; S_out_ and S_in_ represent the corresponding values of in-plane and out-of-plane modes, respectively.

The interfacial phonon scattering across the interface enhanced by the increment in graphene thickness results in a reduction in TBC. Similar phenomena can be observed in research on heat transport across the gra/Al, gra/Ti, and gra/Ni interfaces. To clarify the underlying mechanisms, we calculated the vibrational densities of different modes (in-plane and out-of-plane) of the interfacial copper and graphene layers and obtained the corresponding phonon coupling strengths as shown in Figure 3a, respectively. In this model, the VDOS values of graphene and copper are highly overlapped in the low-frequency region (0–10 THz). The results show that the magnitude of overlap factor S (Figure 3b) in the out-of-plane direction is four times higher than the value in the in-plane direction, so out-of-plane phonon vibration of graphene plays a dominant role in TBC. The phonon coupling strength along the out-of-plane direction monotonically decreases with the increase in the number of graphene layers, which displays a similar trend with TBC.

### 3.2. Effect of the Atomic Structure

**Figure 4:**
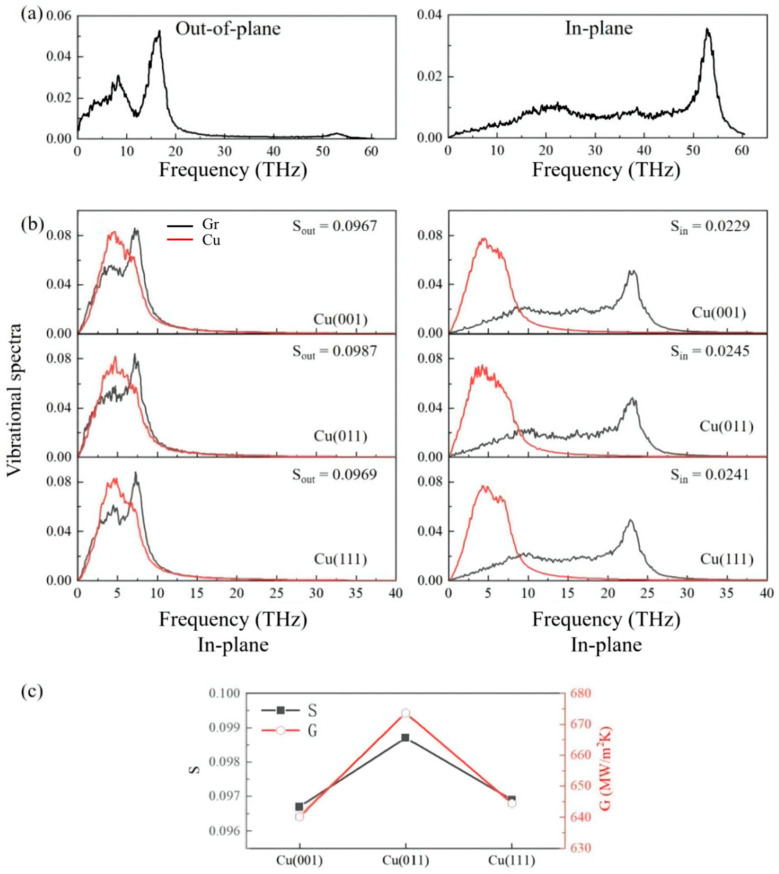
(**a**) Phonon spectra of single graphene at 300 K. (**b**) Out-of-plane and in-plane vibrational spectra of different surfaces. (**c**) The overlap parameter S of the VDOS and corresponding TBC with distinct surfaces.

We create three distinct crystal surfaces—(001), (011), and (111)—of the Cu crystals coupled with graphene at the interface to assess the effect of distinct atomic structures upon thermal transport across the Cu/Gr interface (Figure 1c). To study the influence of atomic structure on thermal transport across the Cu/Gr interface, we construct three different crystal surfaces—(001), (011), and (111)—of the Cu crystals connected with the graphene sheet at the interface (Figure 1c). The variations in the lattice structure inevitably cause the variation in the bonding scheme at the interface. In order to minimize the strain imposed by lattice mismatch, the cross-sectional area (A) was also varied when these three atomic structures were re-matched with monolayer graphene according to their dimensions in the x- and y-directions. The model parameters with distinct crystal planes of Cu can be observed in Table 2. The simulation results at room temperature of 300 K show that the TBC of Cu(011)/Gr (673.61 MW/m^2^K) is about 4.2% and 5.2% higher than the values of the Cu(001)/Gr (640.19 MW/m^2^K) and Cu(111)/Gr (644.41 MW/m^2^K) models, respectively. Figure 4b displays the VDOS values of carbon and copper atoms in graphene and copper layers on diverse contact interfaces. The change curve of S with TBC is depicted in Figure 4c, and the change trend of TBC is in good agreement with S, demonstrating the variety of TBC in various crystal planes. For example, the higher value of S indicates that more low-frequency phonons are activated to promote the interfacial heat transport process. Compared with the VDOS of single graphene (Figure 4a) under the same simulation conditions, the peak densities of the in-plane and out-of-plane state densities of graphene after being compounded with copper moved to the low-frequency region.

**Figure 5:**
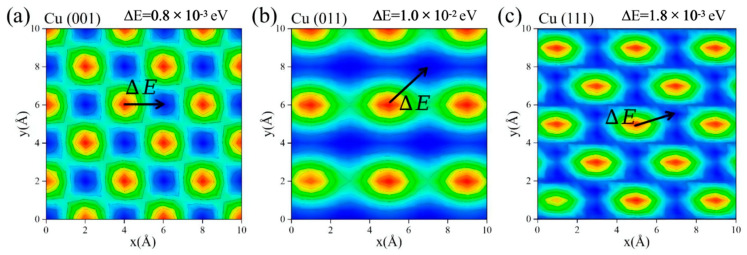
(**a**–**c**) Potential energy distribution contour of adjacent graphene layers with different atomic structures. ΔE represents the difference value between the maximum and minimum potential energy.

We calculated the potential energy distribution on different atomic structures by shifting the graphene layer on the ground in order to further confirm the veracity of the findings. The calculation results are shown in Figure 5. The difference values between the maximum and minimum potential energy of the (001), (011), and (111) surfaces are 0.8 × 10^−3^ eV, 1.0 × 10^−2^ eV, and 1.8 × 10^−3^ eV, respectively. This means that the energy required to move graphene from a stable position to an unstable position is higher on the (011) surface. Therefore, graphene has the strongest binding ability bonding with the (011) surface. Stronger interfacial bonding is good for phonon transmission, as shown in Figure 4b, which indicates that the (011) model possesses the largest S = 0.0987. Therefore, the effect of the atomic structure can be clarified through the potential energy distribution.

**Figure 6:**
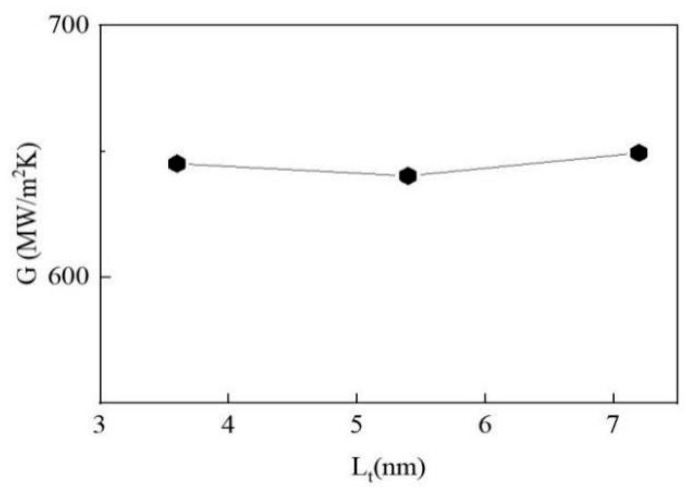
Effect of Cu-bulk thickness on TBC.

We further explore the TBC by elongating the length of the copper matrix, i.e., setting the length of the copper matrix as 3.6 nm, 5.4 nm, and 7.2 nm, respectively. As shown in Figure 6, the TBC is almost independent of the thickness of Cu block L_t_.

### 3.3. Effect of Graphene Vacancy Rate

**Figure 7:**
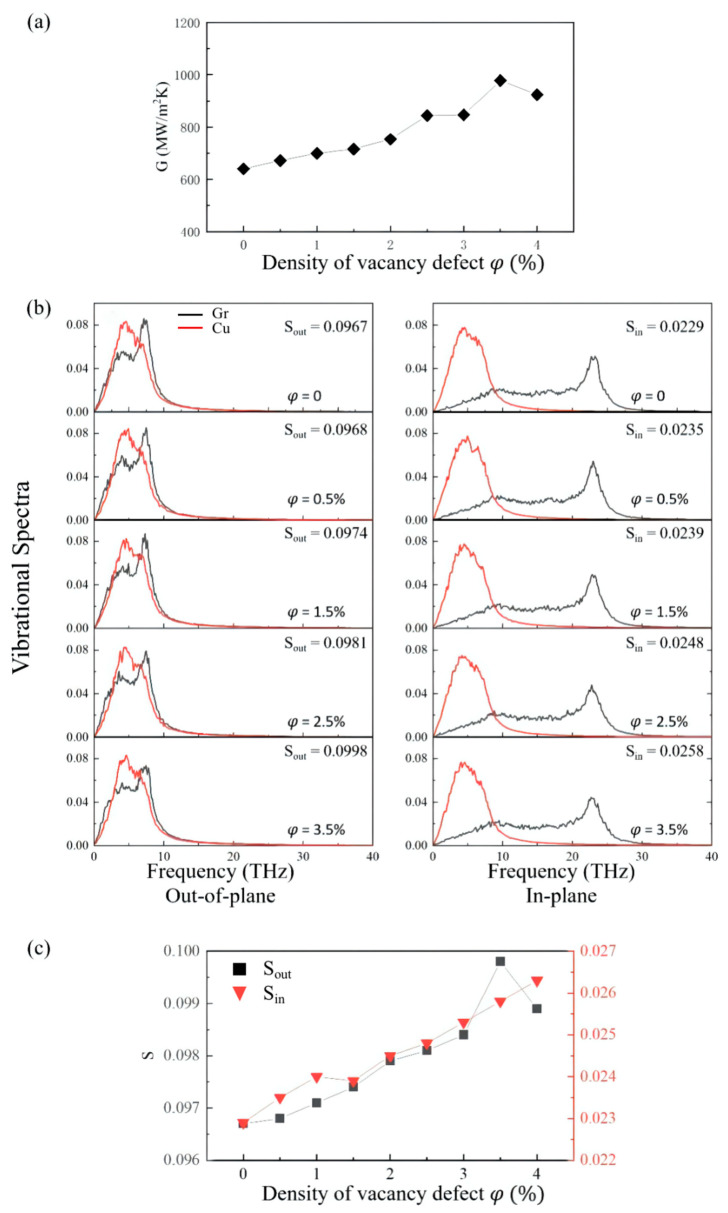
(**a**) Variation in Z-direction interfacial thermal conductance of graphene as a function of graphene defect density. (**b**) Out-of-plane and in-plane VDOS values of Cu and graphene atoms at the interface with diverse density of vacancy defect. (**c**) The overlap of phonon spectrum S with different density of vacancy defect; S_out_ and S_in_ represent the corresponding values of in-plane and out-of-plane modes, respectively.

The influence of the material defects on thermal conductivity cannot be ignored. According to the synthesis approach, there can be diverse types of defects in graphene. In this work, we take the vacancy defect into consideration. The vacancy defect concentrations were sampled from 0 to 4%, and the models were established every 0.5% for simulation calculation. We produce vacancies by randomly removing a given number of carbon atoms from the graphene structure. The results show that, with the increase in the vacancy defect concentration, the interface thermal conductivity of different models fluctuates but shows an overall upward trend. The vacancy defect concentration of the model with the highest interfacial thermal conductivity is 3.5%, and the value is 977 MW/m^2^K, which is 52.6% higher than that of the model without vacancy defect (Figure 7a).

Figure 7b shows the VDOS values of copper and carbon atoms. Previous work has shown that the VDOS values of graphene and copper mainly coincide in the low-frequency region (less than 10 THz). With the introduction of vacancies in graphene, the out-of-plane VDOS of graphene gradually shifts towards the low-frequency region, thus increasing the coupling of the out-of-plane vibration between graphene and copper (Figure 7c). Previous studies have also demonstrated that atoms will lose sp^2^ bonds after removing their neighboring atoms. Consequently, their out-of-plane vibrations will be less constrained than those of pristine graphene and, therefore, possess more low-frequency patterns [40].

## 4. Conclusions

In summary, the interfacial thermal conductivity of the copper–graphene interface was systematically studied by means of MD simulations. The key findings are concluded as follows. First, the thermal conductivity of the interface decreases with the increase in the number of graphene layers. Compared with single-layer graphene, the thermal conductivity of n-layer (*n* > 5) graphene decreases by about 78%. The underlying mechanism is that the increase in graphene layers enhances phonon scattering, which weakens the phonon coupling between copper and graphene in the low-frequency region. Second, Cu(011)/Gr possesses the highest TBC (673.61 MW/m^2^K), which has an increment of 4.2% and 5.2%, compared with the values of the Cu(001)/Gr (640.19 MW/m^2^K) and Cu(111)/Gr (644.41 MW/m^2^K) models, respectively. The effect of atomic structures can be clarified through the potential energy distribution, which indicates that a higher energy barrier within the interface means stronger interfacial bonding and is good for phonon transmission. In addition, the interface thermal conductivity increases gradually when the vacancy defect density of graphene increases from 0 to 3.5%. The increasing trend can be attributed to variation in the vibration frequency schemes caused by the fracture between carbon atoms caused around the vacancy defects.

Compared with previous research regarding the Cu/Gr interface, we have investigated the influence of different factors on the TBC more systematically and gained a comprehensive understanding of the thermal conductance of the copper–graphene interface. The obtained findings are helpful regarding thermal transport mechanisms in future application of Cu/Gr MMCs in thermal management materials.

## Figures and Tables

**Table 1:** The studies of different metal matrix/graphene composites using NEMD method.

Composite	Method	Layer Number	Atomic Structure	Vacancy Rate	Other	Ref.
Cu/Gr	NEMD	√	(011) > (111) > (001)	√	Length of copper matrix	This work
Al/Gr	NEMD	√	(110) > (111) > (100)	×	Strain	[23]
Ti/Gr	NEMD	√	(0001) > ($11\bar{2}0$) > ($10\bar{1}0$)	×	Strain	[24]
Ni/Gr	NEMD	√	×	×	×	[25]

**Table 2:** The model parameters choosing different crystal planes of Cu and the corresponding mismatch strain of graphene.

Crystal Plane	L_x_ (nm)	ε _Gr−x_	L_y_ (nm)	ε _Gr−y_	L_t_ (nm)
Cu(001)	4.69	0.407%	4.69	0.085%	5.42
Cu(011)	2.43	0.57%	4.52	−3.69%	4.98
Cu(111)	2.43	0.57%	5.49	−0.89%	6.04

## Data Availability

The raw data cannot be shared at this time as the data are also part of an ongoing study.
